# Cushing's Syndrome Missed in Pregnancy

**DOI:** 10.7759/cureus.70930

**Published:** 2024-10-06

**Authors:** Aditya Sudarshanan, Rayna M Koshy, Sathia Narayanan Mannath, Cornelius Fernandez, Koshy Jacob

**Affiliations:** 1 Department of Acute Medicine, Pilgrim Hospital, United Lincolnshire Hospitals NHS Trust, Boston, GBR; 2 Department of Internal Medicine, New Cross Hospital, Royal Wolverhampton NHS Trust, Wolverhampton, GBR; 3 Department of Endocrinology and Metabolism, Pilgrim Hospital, United Lincolnshire Hospitals NHS Trust, Boston, GBR; 4 Department of Endocrinology and Metabolism, Eastbourne District General Hospital, East Sussex Healthcare NHS Trust, Eastbourne, GBR

**Keywords:** adrenal adenoma, adrenalectomy, cushing syndrome, gestational diabetes, gestational hypertension, hpa axis in pregnancy, pre-eclampsia, pregnancy

## Abstract

Cushing's syndrome (CS) is uncommon during pregnancy and often difficult to diagnose due to similar symptoms shared with normal pregnancy. This case report discusses a 26-year-old woman who developed CS while pregnant, underlining the importance of early detection and the diagnostic challenges involved. The patient presented with gestational diabetes and pre-eclampsia during pregnancy. Post delivery, she continued to experience hypertension and facial swelling, which led to a diagnosis of CS. The patient underwent a successful laparoscopic adrenalectomy, which normalized her blood pressure and improved her symptoms. This case highlights the need for heightened awareness of CS in pregnant women exhibiting both gestational diabetes and hypertension, as early diagnosis and treatment are essential to reduce maternofoetal complications.

## Introduction

Endogenous Cushing's syndrome (CS) is a rare condition resulting from prolonged excessive cortisol production. The common causes include adrenocorticotropic hormone (ACTH)-producing pituitary adenomas (Cushing’s disease), ectopic ACTH-producing tumours, and cortisol-producing adrenal adenomas [1]. Though cortisol-producing adrenal adenomas are the most common cause of adrenal CS, less common causes include cortisol-producing adrenocortical carcinomas or bilateral micro/macronodular adrenal hyperplasia. Exogenous CS resulting from extended high-dose glucocorticoid treatment is more common than endogenous CS.

CS is rare in pregnancy, with fewer than 250 cases currently published [2]. Clinical diagnosis can be missed due to overlapping symptoms with normal pregnancy. Uncontrolled CS during pregnancy is associated with a significantly higher rate of maternal and foetal complications. If untreated, foetal mortality is nearly 20% and maternal mortality rate is nearly 2% [3], but early diagnosis and appropriate treatment could lower this risk. Despite treatment, CS in pregnancy is associated with significant maternal morbidity, which includes hypertension (40.5%), diabetes mellitus (36.9%), and pre-eclampsia (26.3%). Similarly, it contributes to significant foetal complications, which include preterm birth (43%) and intrauterine growth restriction (21%) [2].

The diagnosis of CS in pregnancy is not straightforward due to various reasons. Firstly, there are many things that are in common between a normal pregnancy and CS in pregnancy, making the diagnosis challenging. Secondly, the currently available tests are not sufficiently validated to differentiate between the physiological hypothalamic-pituitary-adrenal (HPA) axis activation that happens in pregnancy versus pathological hypercortisolemia associated with CS. Finally, there is no consensus regarding the optimal treatment strategy even after CS in pregnancy is diagnosed [3]. Despite all these challenges, accurate diagnosis and treatment of CS in pregnancy are crucial for both the mother and the baby due to significantly raised morbidity and mortality.

The case of a 26-year-old woman presented here highlights the importance of a multidisciplinary team (MDT) approach, involving obstetrics, endocrinology, biochemistry, radiology, and various surgical teams, to ensure comprehensive care for both mother and foetus.

## Case presentation

A 26-year-old primigravida who was previously fit and well and not on any medications presented to the obstetric clinic with mild vaginal bleeding. An ultrasound confirmed early pregnancy (six to eight weeks) and blood tests revealed transient polycythaemia. No obvious cause for vaginal bleeding was found.

A 75-g oral glucose tolerance test (OGTT) was done at 17 and 28 weeks. The OGTT at 28 weeks showed fasting glucose of 4.9 mmol/L and two-hour glucose of 8.4 mmol/L (WHO criteria), suggestive of gestational diabetes mellitus (GDM). Glycosylated haemoglobin (HbA1c) at the time of diagnosis was 31 mmol/mol. Self-monitoring of blood glucose (SMBG) was initiated with dietary modifications and later commenced on metformin. She did not require insulin for the management of GDM.

At 31 weeks, she was found to have raised blood pressure (148/97 mmHg) and was started on labetalol. At 35 weeks, despite being on labetalol 200 mg four times a day and nifedipine 10 mg twice a day, her blood pressure remained high (154/99 mmHg), and urine analysis showed proteinuria. Subsequent 24-hour urine protein was 0.38 grams/day (0-0.15 g/d). There were no severe features of pre-eclampsia. There was no hypokalaemia during the antenatal period.

She underwent an emergency caesarean section at 36 weeks. As the baby was born preterm, the mother and baby were transferred to a tertiary centre immediately post caesarean section and both recovered well. Throughout the antenatal period, the obstetrics team did not notice any obvious clinical features of CS. They might have overlooked it.

Six months postpartum, she was referred to the endocrine team for persistent hypertension and facial swelling, wherein she was found to have easy bruising, purplish abdominal striae, and proximal myopathy. She was on labetalol for hypertension. Her HbA1C was 31 mmol/mol. The comparison of her facial and body features with the photographs taken pre- and post-pregnancy was quite striking.

Investigations

On investigation, 24-hour urinary free cortisol levels were more than three times the upper limit of normal at 1684, 1923, and 1866 nmol/24 hours (55-260 nmol/24 hours) on three consecutive days, indicating significantly elevated urinary free cortisol levels suggestive of CS (Table 1). A 1 mg overnight dexamethasone suppression test was unsuppressed with cortisol of 641 nmol/L. This was followed by a low-dose dexamethasone suppression test, which failed to suppress at 590 nmol/L with an ACTH of 9 ng/L, confirming the diagnosis of ACTH-independent CS.

**Table 1:** Summary of adrenal hormone profile.

Test	Result	Normal range
24-hour urinary free cortisol	1684, 1923, and 1866 nmol/24 hours	10-147 nmol/24 hours
1 mg overnight dexamethasone suppression test	Cortisol: 641 nmol/L	-
Low-dose dexamethasone suppression test	Cortisol: 590 nmol/L	-
Adrenocorticotropic hormone (ACTH)	ACTH: 9 ng/L	ACTH: 0-46 ng/L
24-hour urinary fractionated metanephrines	Normal	Normetadrenaline: 0-3000 nmol/24 hours; metadrenaline: 0-1000 nmol/24 hours; 3-methoxytyramine: 0-2300 nmol/24 hours
Serum testosterone	<0.7 nmol/L	0-2.9 nmol/L
Dehydroepiandrosterone sulphate (DHEA-S)	0.8 μmol/L	0.9-11.7 μmol/L

**Figure 1:**
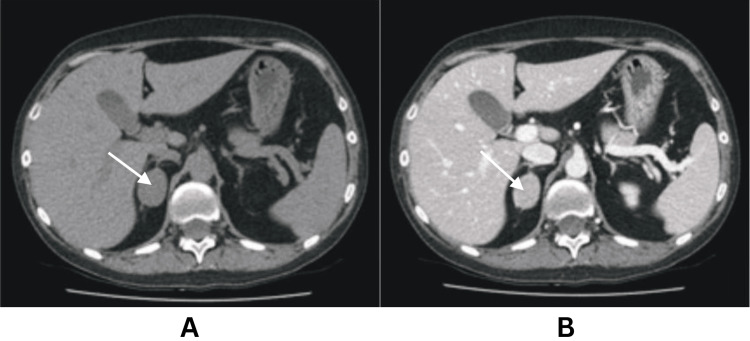
CT of the abdomen and pelvis showing right adrenal adenoma. Panel A illustrates the pre-contrast image with 40±15 HU. Panel B illustrates the post-contrast image.

CT of the abdomen and pelvis revealed a 38 x 34 x 25 mm nodular mass in the right adrenal gland representing an adrenal adenoma (Figure 1). A 24-hour urinary fractionated metanephrines testing was normal (normetadrenaline = 773 (0-3000), metadrenaline = 348 (0-1000), and 3-methoxytyramine = 956 (0-2300) nmol/24 hours, respectively). Serum testosterone of <0.7 (0-2.9) nmol/L, and dehydroepiandrosterone sulphate (DHEA-S) of 0.8 (0.9-11.7) μmol/L ruled out adrenal androgen excess.

Management

**Figure 2:**
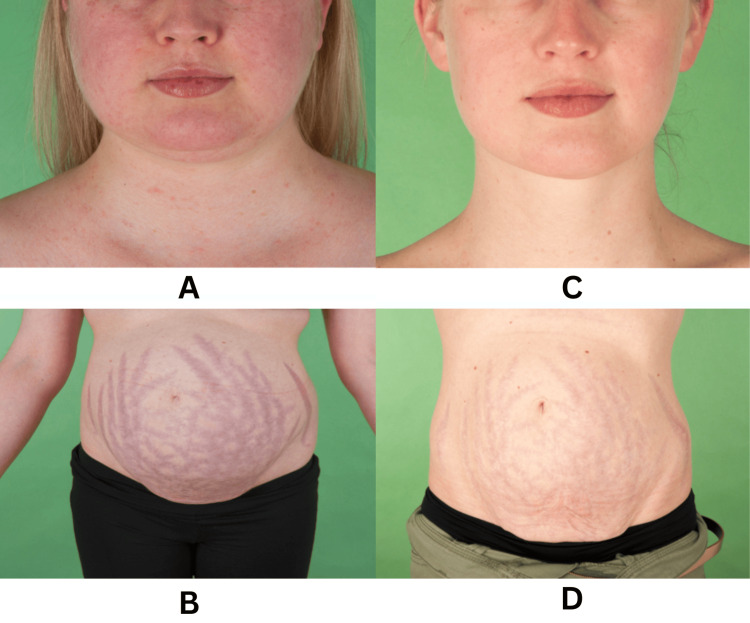
Panels A and B illustrate pre-operative facial and abdominal appearance. Panels C and D illustrate improved facial and abdominal appearance four months post surgery.

The patient was initially treated with a steroidogenesis inhibitor (ketoconazole) and subsequently underwent laparoscopic right adrenalectomy. The histology confirmed a benign adrenocortical adenoma. Following the right adrenalectomy, there was remission of hypertension along with noticeable improvement in her facial and body features, as illustrated in Figure 2.

Follow-up

She developed post-operative HPA axis suppression and was treated with hydrocortisone, which was gradually tapered under close monitoring with cortisol day curves. While on hydrocortisone, 18 months post adrenalectomy, she became pregnant for the second time. She continued hydrocortisone 5 mg once a day throughout this pregnancy, with adherence to sick-day rules. A 75-g OGTT during this pregnancy did not show GDM on two occasions (fasting glucose of 4.3 and 3.8, respectively, and a two-hour value of 4.2 and 5.2 mmol/L, respectively). Moreover, blood pressure remained normal throughout. Post delivery, she had a short synacthen test, which revealed optimal adrenal reserve, and hydrocortisone was discontinued nearly three years after surgery. Since then, the patient has remained asymptomatic and free of any medications related to adrenals.

## Discussion

HPA axis in pregnancy

Anovulatory infertility is common in women with untreated CS. Though many successful pregnancies are reported in women with CS, these might be accompanied by increased morbidity and mortality to the foetus. Owing to the HPA axis physiological changes that happen during pregnancy, diagnosing CS is a challenge during that time. However, early diagnosis and therapy are crucial to limit the complications for both the mother and the foetus [4].

Normal human gestation significantly impacts the maternal HPA axis. Rising placental oestrogen that accompanies pregnancy boosts the hepatic production of corticosteroid-binding globulin (CBG), leading to elevated circulating bound cortisol levels and a transient drop in free cortisol. However, the free cortisol levels also rise progressively during pregnancy, reaching levels similar to those in CS [5]. Plasma ACTH levels increase alongside cortisol. The exact reasons for this ACTH rise are unclear, but it is hypothesized that the transient drop in free cortisol due to increased CBG triggers increased ACTH stimulation, leading to subsequent cortisol production with raised bound and free cortisol levels [6]. Free cortisol levels remain raised in trimesters two and three, and peaking during labour [7].

Elevated free cortisol levels during pregnancy, especially in the later stages, may be attributed to several factors, including the anti-glucocorticoid effects of progesterone, alterations in the negative feedback mechanisms regulating ACTH secretion, or autonomous production of ACTH by the placenta [6]. Plasma ACTH levels rise throughout pregnancy and peak during labour, with potential contributions from both pituitary and placental sources. Placental-derived ACTH may play a significant role in hypercortisolism during pregnancy [7]. Urocortins, which are members of the corticotropin-releasing hormone (CRH) peptide family, also participate in the activation of the HPA axis. Urocortin 1, expressed in the human placenta, may contribute to the sustained activation of the HPA axis in late pregnancy [8]. The HPA axis response to exogenous glucocorticoids is blunted during pregnancy, with reduced suppression of cortisol levels after dexamethasone administration.

In early pregnancy, the foetus is protected from hypercortisolemia happening in the mother due to the presence of the 11β-HSD2 (11β-hydroxysteroid dehydrogenase 2) enzyme, which is found in the syncytial trophoblastic cells. This enzyme, due to its ability to convert cortisol into cortisone and corticosterone into 11-dehydrocorticosterone, ensures that foetal active glucocorticoid levels are significantly lower than maternal cortisol levels [8].

Morbidity and mortality

Nearly 70% of pregnancies with CS exhibit increased morbidity and mortality risk to the mother. The main issues include hypertension, diabetes mellitus, and prediabetes. Other issues include impaired healing of wounds, osteoporosis, fractures, psychiatric manifestations, heart failure, and mortality. There is also an increased risk of spontaneous abortion, perinatal mortality, premature birth, and intrauterine growth restriction. Foetal adrenal insufficiency is rare [9].

Causes of CS in pregnancy

The causes of CS in pregnancy differ from non-pregnant women. ACTH-independent CS from adrenal adenomas accounts for about 40% to 50% of CS in pregnant women, in comparison to nearly 15% in non-pregnant women [10]. The higher incidence of adrenal CS in pregnancy is not fully understood but may be due to lower ovulation rates in women with Cushing’s disease compared to those with adrenal CS. Women with ectopic adrenocorticotrophic hormone secretion experience severe hypercortisolemia and amenorrhea. This likely explains its reduced prevalence in pregnancy. The interference in reproductive function is less pronounced with pure cortisol-secreting adrenal adenomas compared to the mixed cortisol and androgen excess produced by adrenocortical carcinomas or ACTH-dependent adrenal hyperplasia. This is reflected in the lesser degree of hypercortisolism and mild HPA axis suppression observed with adrenal adenomas [11].

Screening and diagnosis

In non-pregnant women, screening for CS involves identifying increased cortisol production (urinary free cortisol or UFC), a disrupted diurnal rhythm (late-night salivary cortisol or LNSC), or reduced cortisol suppression (dexamethasone suppression test). However, normal pregnancy-related changes in the HPA axis, including increased CBG, increased plasma cortisol, increased ACTH, two- to three-fold rise in free cortisol, and a blunted HPA axis response to exogenous glucocorticoids, complicate CS screening [12]. In non-pregnant women, UFC levels more than three times the normal range are indicative of CS, whereas, in pregnancy, though UFC remains unaffected in the first trimester, it can increase to threefold of normal in later trimesters. Hence, UFC values only above four-fold the upper limit of normal are indicative of CS in pregnancy.

Dexamethasone suppression tests are less reliable in pregnancy due to a higher risk of false-positive results, limiting their diagnostic utility. The standard screening tests for CS are prone to higher false-positive diagnoses in pregnant women unless cut-off values specific to pregnancy are used for the UFC and dexamethasone suppression test. The midnight serum cortisol (MNSC) measurements are accurate in confirming an altered circadian rhythm with the cut-off being sleeping serum cortisol >1.8 mcg/dL (50 nmol/L) or non-sleeping serum cortisol >7.5 mcg/dL (207 nmol/L) [12].

Recent studies suggest that a three-fold increase above the normal upper limit of LNSC (>12 nmol/L) has 100% sensitivity and specificity for diagnosing overt CS in non-pregnant women [13]. This cut-off is also applicable to pregnant women. The LNSC threshold values for pregnancy are defined as <6.9 nmol/L at trimester one, <7.2 nmol/L at trimester two, and <9.1 nmol/L at trimester three [14]. LNSC has the highest reliability for diagnosing CS during pregnancy. ACTH levels may fail to suppress even in women with adrenal CS. This results from the stimulatory influence of CRH of placental origin on the mother's pituitary. This may hinder the localization (ACTH-dependent versus ACTH-independent CS).

Adrenal Imaging

Ultrasound imaging is generally safe and effective but less sensitive for smaller adrenal tumours, necessitating additional imaging modalities. MRI and CT scans are used effectively, with MRI being preferred during pregnancy due to the absence of ionizing radiation risk [1].

Pituitary Imaging

MRI is not routinely used in pregnant women due to safety concerns. It is contraindicated in the first trimester due to potential (though unproven) teratogenic effects but is considered safe after 32 weeks [1]. Starting from 12 weeks up to 32 weeks, the disadvantages of having an MRI should be weighed against the advantages, recognizing that incidental tumours (≤ 6 mm) can appear in as high as 10% of the normal population. Physiological enlargement of the pituitary gland reaching two-fold the normal size by trimester three possibly leads to more incidentalomas being identified during pregnancy compared to non-pregnant individuals.

In a nutshell, a non-contrast MRI is the preferred imaging for localizing hypercortisolemia during pregnancy. However, a non-contrast pituitary MRI might fail in detecting a microadenoma, as the pituitary gland enlarges during pregnancy, making it difficult to distinguish between a pathological lesion and physiological enlargement. If an adrenal CS is a possibility, an MRI of the abdomen is the recommended imaging.

Treatment

Reducing the UFC excretion to levels seen in normal pregnancies may help prevent adverse outcomes. However, treatment for pregnant patients with CS is often sporadic and initiated late in pregnancy, making it difficult to assess its efficacy.

In patients with confirmed hypercortisolism, treatment should be personalized to minimize foetal loss risk. Managing comorbid conditions like diabetes mellitus and hypokalaemia is important. Hypertension in pregnant women with CS is treated with drugs such as nifedipine, amlodipine, and methyldopa.

Surgical resection of adrenal and pituitary adenomas is typically postponed until the later second trimester. Surgical resection during first-trimester surgical intervention is generally avoided due to the higher risk of foetal loss.

If CS is diagnosed early in pregnancy, metyrapone (250-750 mg/day in three divided doses) is preferred to reduce serum cortisol to acceptable levels for pregnancy. Metyrapone is generally well-tolerated and has not shown adverse effects on maternal hepatic function or foetal development, though with higher doses, there is a potential for exacerbating hypertension, pre-eclampsia, and hypokalaemia due to 11-deoxycorticosterone accumulation. Moreover, prolonged use can occasionally lead to hypocortisolaemia. Although metyrapone can cross the placenta, its impact on foetal steroid production is minimal [1].

Use of ketoconazole is limited to severe hypercortisolemia due to potential maternal hepatotoxicity and theoretical risks of feminizing a male foetus, though feminization is not observed in practice. Other drugs like mitotane, etomidate, and mifepristone are severely teratogenic [1].

In women with CS in whom metyrapone was used throughout pregnancy, the drug should be withdrawn for a while before labour and resumed after delivery. All pregnant women with CS, regardless of their treatment (pharmacological or surgical), should receive intrapartum hydrocortisone intravenously. Hydrocortisone can then be slowly reduced and eventually discontinued [1].

What challenges are associated with diagnosing CS in pregnancy?

Adrenal CS contributes to almost half of all CS cases during pregnancy. False-positive results in the overnight dexamethasone suppression test (ODST) can occur due to a blunted HPA axis response to dexamethasone. UFC values exceeding four times the upper limit of normal strongly suggest CS in pregnancy. The most reliable screening tests for CS in pregnancy are the LNSC and MNSC. Elevated ACTH levels in pregnancy can hinder the localization of CS, making it difficult to differentiate between ACTH-dependent and ACTH-independent CS. The management of CS in pregnancy is tailored to the trimester of diagnosis and the severity of the condition.

## Conclusions

This case report highlights the critical need for maintaining a high level of suspicion for CS in pregnant women, especially those presenting with both gestational diabetes and hypertension. Early diagnosis and treatment are vital to minimize potential complications for both the mother and the foetus. The diagnostic process is complex, as physiological changes in the HPA axis during pregnancy can resemble the symptoms of CS. Therefore, a thorough approach, including clinical evaluation, biochemical tests, and imaging studies, is crucial for accurate diagnosis and timely management.
